# Opposing Nodal and BMP Signals Regulate Left–Right Asymmetry in the Sea Urchin Larva

**DOI:** 10.1371/journal.pbio.1001402

**Published:** 2012-10-09

**Authors:** Yi-Jyun Luo, Yi-Hsien Su

**Affiliations:** Institute of Cellular and Organismic Biology, Academia Sinica, Nankang, Taipei, Taiwan; Osaka University, Japan

## Abstract

Nodal and BMP signals are important for establishing left-right (LR) asymmetry in vertebrates. In sea urchins, Nodal signaling prevents the formation of the rudiment on the right side. However, the opposing pathway to Nodal signaling during LR axis establishment is not clear. Here, we revealed that BMP signaling is activated in the left coelomic pouch, specifically in the *veg2* lineage, but not in the small micromeres. By perturbing BMP activities, we demonstrated that BMP signaling is required for activating the expression of the left-sided genes and the formation of the left-sided structures. On the other hand, Nodal signals on the right side inhibit BMP signaling and control LR asymmetric separation and apoptosis of the small micromeres. Our findings show that BMP signaling is the positive signal for left-sided development in sea urchins, suggesting that the opposing roles of Nodal and BMP signals in establishing LR asymmetry are conserved in deuterostomes.

## Abstract 2

A left-right patterning study in developing sea urchin shows that the opposing roles of Nodal and BMP signaling in patterning the left-right axis are conserved in deuterostomes.

## Author Summary

Bilateral animals that are externally symmetric in appearance often have internal organs that are asymmetric with respect to the left and right sides of their bodies. Two signals, Nodal and BMP, have been shown to establish this asymmetry during vertebrate embryogenesis. We investigate here whether the same mechanisms that establish left-right patterning in vertebrates are conserved in invertebrate animals, specifically in the California purple sea urchin. This sea urchin passes through various stages in its lifecycle before developing to adulthood, including a feeding larva stage in which the tissue that goes on to form the adult, the so-called adult rudiment, forms on the left side. Previous studies have shown that right-sided Nodal signaling in sea urchins prevents the formation of the adult rudiment. In this study, we show that BMP signaling is activated on the left side and is required for the development of this left-sided structure. We also demonstrate that Nodal signaling blocks BMP activity and induces apoptosis of the tissue that forms the adult rudiment on the right side. We propose that the roles of Nodal and BMP signals in establishing left-right asymmetry are evolutionarily conserved.

## Introduction

One of the most fascinating features of bilaterian development is the consistent left-right (LR) asymmetry of their internal organs. In the past two decades, studies of molecular pathways controlling LR asymmetry have broadened our knowledge regarding the dissimilar and conserved mechanisms among different animal models [1]. It has been suggested that the mechanisms of initial symmetry breaking are not conserved across different vertebrate classes [2]. For example, a leftward fluid flow generated by the posteriorly tilted nodal cilia initiates left-sided gene expression in the mouse embryo [3]. On the other hand, differential activities of ion pumps in early stage embryos are important for establishing LR asymmetry in the chick, frog, and zebrafish [4]. Although detailed symmetry-breaking mechanisms vary among different vertebrate species, the common outcome is the expression of *nodal*, which encodes a transforming growth factor β (TGFβ) ligand, in the left lateral plate mesoderm (LPM) [5]. The Nodal pathway not only regulates LR asymmetry in vertebrates but also controls the formation of the left-sided adult rudiment in sea urchins [6] and body chirality in snails [7].

Bone morphogenic protein (BMP), another TGFβ family member, is also involved in LR patterning [8]. In opposition to conserved *nodal* expression on the left side, BMP transcripts or activities are observed on the right side of the node or LPM. For example, *bmp4* is expressed on the right side of Hensen's node in the chick embryo and initiates a right-sided signaling cascade [9]. Although *bmp* genes are expressed symmetrically in the LPM of the mouse and chick embryos, BMP activity is greater on the right side due to the presence of BMP antagonists on the left side [10]–[13]. A BMP/ALK2/Smad-mediated signaling pathway is also proposed to be active on the right side of the *Xenopus* embryo [14]. Therefore, right-sided BMP opposing left-sided Nodal seems to be a conserved feature. The role of BMP in LR patterning is largely unknown in invertebrates, even though right-sided expressed *dpp-bmp2/4* plays important roles in shell formation and coiling in gastropods [15],[16].

Sea urchins belong to the phylum Echinodermata, which is characterized by a pentaradiate adult body plan. In indirect developing sea urchins, the adults are derived from bilaterally symmetric larvae. The transition from a bilaterally symmetric to pentasymmetric body plan relies on a LR asymmetrical control that results in the formation of an adult rudiment on the left side of the larva (Figure S1). During gastrulation, a coelomic pouch (CP) composed of *veg2* mesoderm and small micromeres (Smm) forms at the archenteron tip and later separates into left and right pouches in the bilateral gastrula [17]. The first morphological signature of LR asymmetry in the pluteus larva is the extension of a duct-like structure, the hydroporic canal (HC), from the left CP to the aboral ectoderm where the hydropore forms [18]. The ciliated HC is thought to be an excretory organ that contributes to normal body width maintenance in the larva [19],[20] and later differentiates into a portion of the adult water vascular system [21]. The differentiated left coelom together with the invaginated left oral ectoderm, called the primary vestibule, develop into an adult rudiment with pentaradial symmetry [22],[23].

Our knowledge of the molecular mechanisms concerning LR patterning in sea urchins is relatively limited. It has been shown that sea urchin LR axis specification depends on cell interactions [24]. A series of microsurgery experiments revealed that the positioning of the adult rudiment on the left side is directed by signals from the right side [25]. Duboc et al. further demonstrated that *nodal* expression on the right side, which is reversed when compared to vertebrates, prevents the formation of the adult rudiment [6]. In addition, signals emitted from the micromeres also regulate LR asymmetry [26], although the identity of the micromere-derived signal remains unknown. It is also not known whether positive signals or a default pathway are required for the left-sided structure development.

In this study, we focused on the role of the BMP pathway and examined the molecular basis of LR asymmetry in the sea urchin embryo. We found that *bmp* genes are symmetrically expressed in skeletogenic micromeres, but BMP signaling is asymmetrically activated in the left CP-derived HC. Through cell lineage analysis, we detected active BMP signaling in *veg2* descendants but not in the Smm. We further provided evidence that BMP signaling is required for left-sided structure development and the expression of several left-sided marker genes. We also demonstrate that right-sided Nodal signaling restricts BMP activity and is involved in the asymmetrical separation and apoptosis of the Smm. We discuss these findings in the context of Nodal and BMP signaling in patterning LR asymmetry in the sea urchin embryo.

## Results

### pSmad1/5/8 Was Detected on the Left Side of the Larva

**Figure 1:**
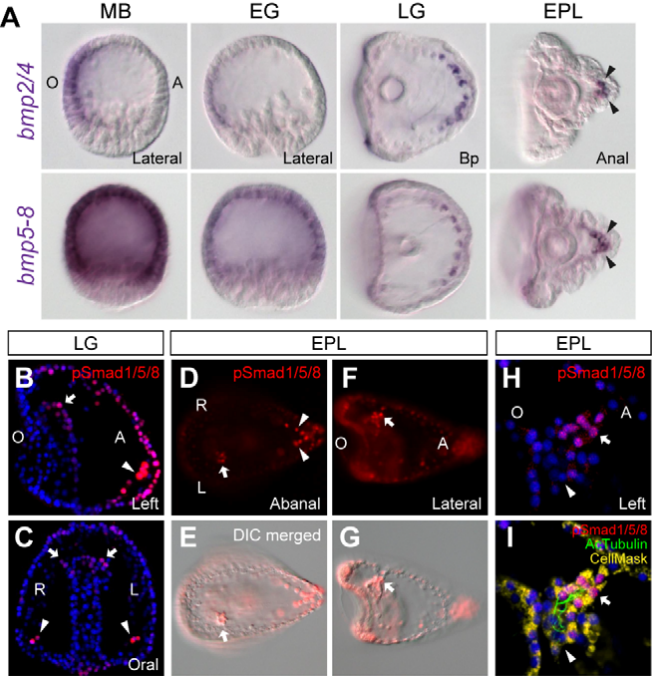
Expression patterns of *bmp* genes and pSmad staining during sea urchin development. (A) ISH of *bmp2/4* and *bmp5–8* in the mesenchyme blastula (MB), early gastrula (EG), late gastrula (LG), and early pluteus (EPL). Immunostaining of pSmad in the LG (B and C) and EPL (D–I) stages. In (A–G), the arrowheads indicate the bilateral signals in the aboral skeletogenic cells, and the arrows indicate signals in the CPs. (H–I) Higher magnifications of the left CP show pSmad staining in the ciliated HC (arrow) but not in the base (arrowhead). An antibody against acetylated α-tubulin antibody (AcTubulin) was used to stain cilia. The observed view is indicated at the bottom right corner of each panel (Bp, blastopore view), and the axes are labeled as O, oral; A, aboral; L, left; and R, right.

To study the role of BMP signaling in LR asymmetry in sea urchins, we first examined the expression patterns of genes related to the BMP signaling pathway. The sea urchin genome contains three *bmp* ligand genes: *bmp2/4*, *bmp3*, and *bmp5–8* [27]. *Bmp2/4* is initially transcribed in the oral ectoderm at the blastula stage, but the Bmp2/4 ligand translocates to the aboral side and plays key roles in the aboral ectoderm gene regulatory network [28]–[33]. The expression patterns of sea urchin *bmp3* and *bmp5–8* have not been elucidated. Therefore, we performed quantitative PCR and discovered that the *bmp3* transcripts are not detectable during the first 3 d of development, whereas *bmp5–8* was expressed in the egg and during this period (unpublished data). In situ hybridization demonstrated that *bmp2/4* expression shifted from the oral ectoderm to the aboral skeletogenic mesenchyme cells during gastrulation and remained in a few cells at the apex of the pluteus larva (Figure 1A). This expression pattern is similar to *Pl-bmp2/4* from sea urchin *Paracentrotus lividus*; however, the timing for the expression domain shift occurs later in this species because its oral ectodermal expression can still be observed in the gastrula stage [27],[31]. The *bmp5–8* transcripts were ubiquitously detected in the egg and later in the whole ectoderm at the blastula and early gastrula stages. Similar to *bmp2/4*, *bmp5–8* expression also shifted to the aboral skeletogenic cells at the late gastrula and pluteus stages (Figure 1A). Both the *bmp2/4* and *bmp5–8* genes were bilaterally expressed during all analyzed stages. We further examined the expression patterns of BMP receptors (*alk1/2*, *alk3/6*, *bmpr2*, and *acvr2*) and did not observe asymmetrical LR expression (unpublished data). We then investigated BMP signaling activity by monitoring the phosphorylation and nuclear translocation of its downstream mediator Smad1/5/8 (pSmad). Immunostaining with a pSmad antibody showed that BMP signaling was activated in the aboral ectoderm at the blastula stage, as previously reported [29],[31]. During gastrulation, pSmad was bilaterally detected in the skeletogenic, ectodermal, and archenteron cells on the aboral side of the embryo (Figure 1B,C). In addition, we observed an LR asymmetric pSmad pattern with a stronger signal on the left CP of the pluteus larva (Figure 1D–G). Detailed observation revealed that the staining was restricted to the HC but was absent at the base of the left CP (Figure 1H,I). These results suggest that although *bmp2/4* and *bmp5–8* are symmetrically expressed, BMP signaling is activated on the left side in the pluteus larva and may play a role in controlling left-sided development in sea urchins.

### pSmad-Positive Cells in the Left CP Are *veg2* Descendants

**Figure 2:**
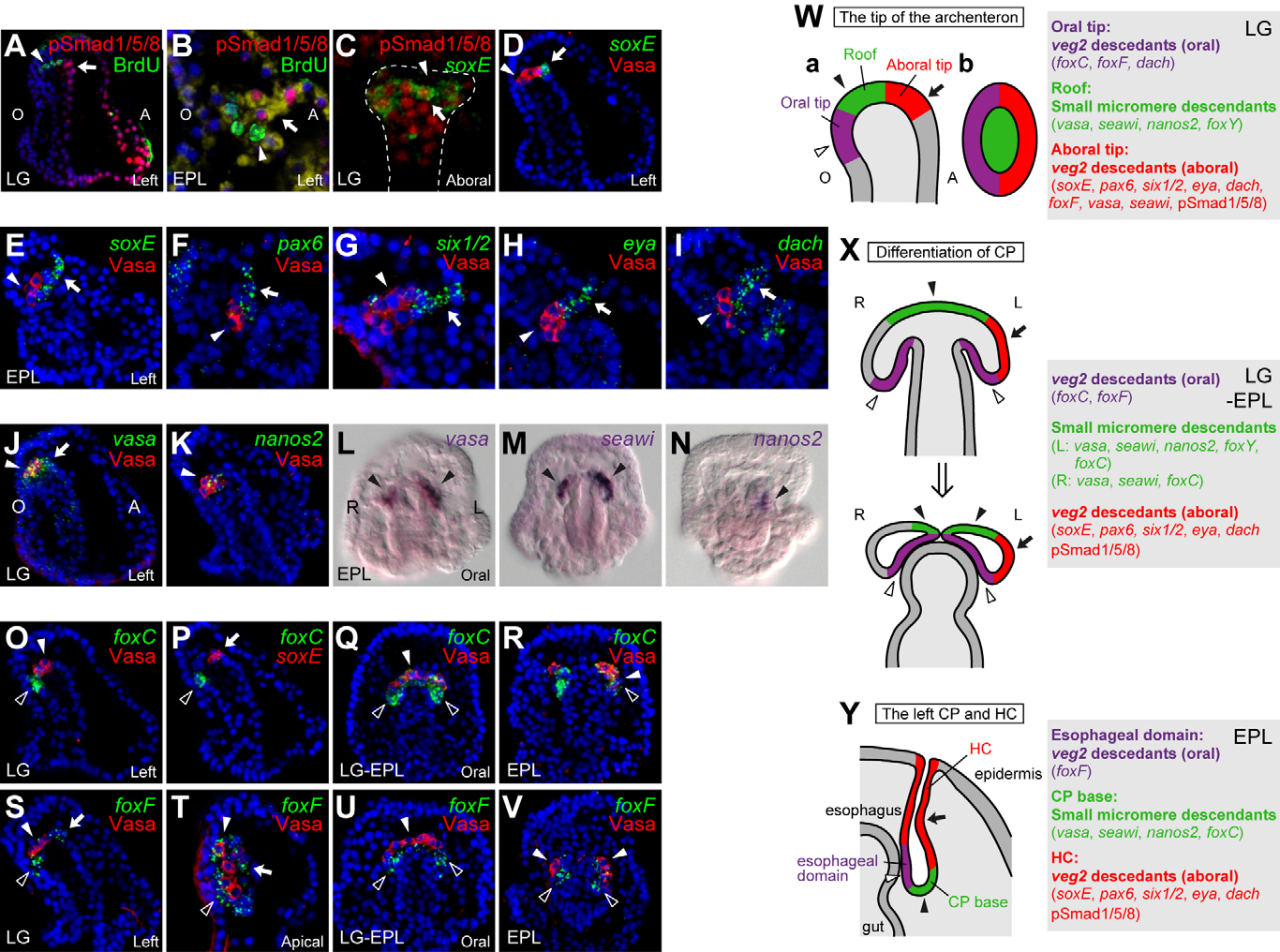
Cell lineages and gene expression domains in the archenteron tip and the left CP. BrdU-labeled Smm and pSmad staining at the LG (A) and EPL stage (B; higher magnification of the left CP). (C) Double staining for *soxE* transcripts and pSmad in the archenteron tip (marked by the dotted line) at the LG stage. ISH of *soxE* and Vasa protein staining at the LG (D) and EPL stages (E). (F–I) Double staining of Vasa protein and ISH of *pax6*, *six1/2*, *eya*, and *dach* at the EPL stage. Expression patterns of germline marker genes at the LG (J and K) and EPL (L–N) stages. ISH of *foxC* (O–R) and *foxF* (S–V) at the LG, the intermediate LG-EPL, and EPL stages. Illustrations of gene expression domains in the archenteron tip at the LG stage (W), the intermediate LG-EPL stage showing the CP dividing process (X), and the left CP at the EPL stage (Y). The embryos were viewed from the left (Wa), apical (Wb), or oral side (X and Y). The arrows indicate *veg2* descendants localized at the aboral tip of the archenteron at the LG stage and the HC at the EPL stage. The open arrowheads denote *veg2* lineage at the oral tip of the archenteron at the LG stage and the esophageal domain at the EPL stage and the solid arrowheads indicate the Smm.

The CPs consist of two cell lineages: Smm and *veg2* descendants [34]. Because only some cells in the left CP were labeled with pSmad, we examined the lineage of these cells by using BrdU pulse-chase labeling at the one-cell stage to mark the slow cell-cycling Smm [35]. At the gastrula stage, BrdU-labeled Smm were located in the roof of the archenteron, while the aboral side of the archenteron tip was stained with pSmad in a graded manner from the tip to the base (Figure 2A). Later at the pluteus stage, pSmad was detected in the cells constituting the HC and did not overlap with the BrdU-labeled Smm (Figure 2B). These results indicate that the pSmad-positive cells in the left CP were derived from the *veg2* cells and differentiated into the HC. Smm lacking pSmad staining were located in the roof of the archenteron at the gastrula stage and later constituted the base of the left CP in the pluteus larva.

### Differential Gene Expression at the Archenteron Tip and the Left CP

Several genes are known to be expressed at the tip of the archenteron during gastrulation and later in the CPs, but the cell lineages in which these genes are expressed are unknown. Therefore, we examined the expression patterns of several known left-sided markers in conjunction with pSmad staining that labeled *veg2* descendants or a *Drosophila* Vasa antibody that labeled Smm similarly to BrdU (Figure S2A) [36]. We detected *soxE* transcripts [6], at the gastrula stage on the aboral side of the archenteron tip (aboral tip) where pSmad staining was strongest (Figure 2C) and Vasa protein was absent (Figure 2D). At the pluteus stage, *soxE* was expressed in the HC of the left CP but not in Vasa-positive Smm (Figure 2E), which was similar to the pSmad pattern. Comparable expression patterns were observed for the left-sided markers *pax6*, *six1/2*, and genes encoding Six1/2 cofactors *eya* [38] and *dach* (Figure 2F–I). However, *dach* was also expressed on the oral side of the archenteron tip at the late gastrula stage (Figure S2B,C).

We also reexamined germ-line-associated genes known to be expressed in the Smm, such as *vasa*, *seawi*, and *nanos2* [37]. Indeed, these genes were expressed in Vasa protein-positive Smm located in the archenteron roof at the gastrula stage (Figure 2J,K). *Vasa* and *seawi* transcripts were also detected on the aboral side of the archenteron tip (Figure S2D, arrows). At the pluteus stage, *vasa* and *seawi* continued to be expressed in the Smm in both CPs, although *nanos2* expression quickly disappeared on the right side (Figure 2L–N). We also noted that *vasa* and *seawi* mRNA signals were stronger on the left side.

Three forkhead genes, *foxC*, *foxF*, and *foxY*, were reported to be expressed in the archenteron tip during gastrulation and later in the CPs [39]–[41]. We detected *foxC* transcripts on the oral side of the archenteron tip at the gastrula stage (Figures 2O and S2E). This expression pattern marked a novel domain (oral tip) that is not derived from the Smm that contain Vasa protein and *nanos2* transcript and does not express *soxE* (Figures 2P and S2F). As the CP budded out from the archenteron tip and separated into the left and right CP, *foxC* expression decreased in the oral tip and was detected in the Smm (Figures 2Q,R and S2F). The *foxF* expression pattern was broader than that of *foxC* at the gastrula stage. In addition to the expression in the oral tip, *foxF* was also weakly expressed in the aboral tip (Figure 2S,T). At the pluteus stage, *foxF* transcripts remained in the oral tip cells that constitute part of the CPs adjacent to the esophagus (esophageal domain) (Figures 2U,V and S2F). The third forkhead gene, *foxY*, was expressed in the Smm from the blastula stage to the gastrula stage; however, transcripts were not detected at the pluteus stage (Figure S2G).

Collectively, there are at least three domains on the archenteron tip at the gastrula stage: the *veg2*-derived oral and aboral tips and the Smm located on the roof (Figure 2W). Each of these three domains expressed a set of genes. *FoxC*, *foxF*, and *dach* were expressed in the oral tip; the aboral tip contained pSmad signal and expressed *soxE*, *pax6*, *six1/2*, *eya*, *dach*, *vasa*, *seawi*, and *foxF*; and the roof contained Vasa protein and expressed *vasa*, *seawi*, *nanos2*, and *foxY*. During CP formation, the pSmad signal on the right side disappeared. After the two CPs separated, the left CP was distinguishable from the right CP based on the pSmad signal and the larger expression domains of *vasa* and seawi (Figure 2X). At the pluteus stage, the left CP could also be distinguished into three domains based on gene expression patterns (Figure 2Y). The HC was derived from the left aboral tip of the archenteron and was marked with pSmad and expressed the same set of genes, except *vasa*, *seawi*, and *foxF*. The cells at the oral tip of the archenteron moved to a position that lay next to the esophagus and continued to express *foxF*. These cells were at the same location as the myoblasts that were labeled with an anti-actin antibody, and they may have later extended processes and differentiated into esophageal muscles [42]. The Smm located in the roof of the archenteron at the gastrula stage moved to the base of the CP in the pluteus larva, which represented the third domain that expressed *foxC* and germline markers *vasa*, *seawi*, and *nanos2*.

### BMP Signaling Is Required for Left-Sided Structure Formation and Gene Expression in *veg2* Descendants

**Figure 3:**
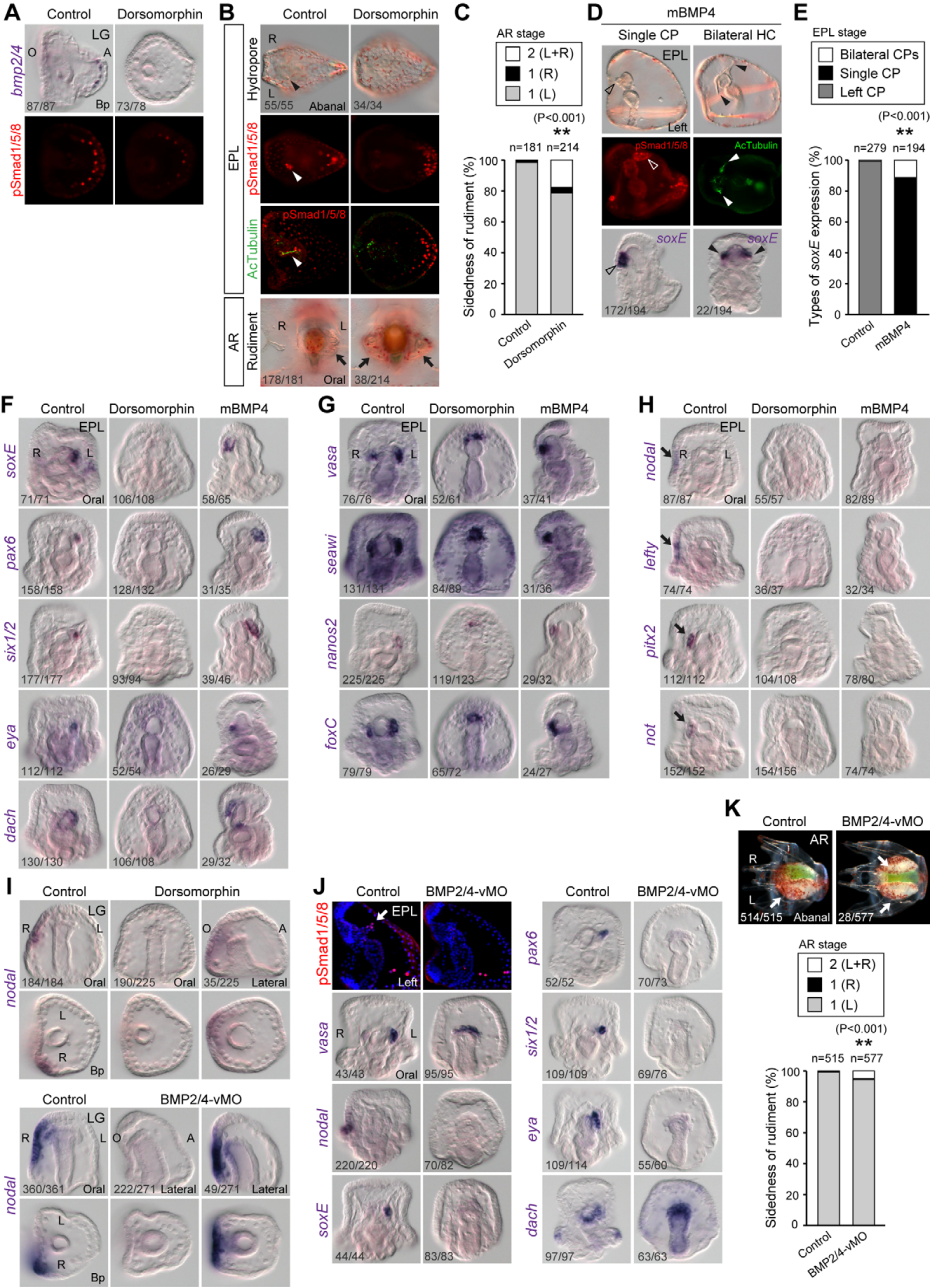
BMP is required for left-sided structure formation and gene expression in the aboral *veg2* descendants. ISH of *bmp2/4* and pSmad immunostaining in control and DM-treated embryos at the LG (A, treated from 24 to 48 hpf) or EPL stage (B, treated from 42 to 48 hpf). The arrowheads in (B) indicate the single left HC. The sides of the rudiments (arrows) were observed at the advanced rudiment (AR) stage (B, bottom panels). (C) The ratios of rudiment sidedness in control and DM-treated larvae (χ*^2^* test). (D) Immunostaining for pSmad or acetylated α-tubulin and ISH for *soxE* in mBMP4-treated embryos. The open arrowheads denote the single undivided CP, and the arrowheads indicate bilateral HCs. (E) The ratios of *soxE* expression patterns (χ*^2^* test). (F–H) ISH analyses of LR marker genes in BMP signaling-perturbed embryos. The arrows in (H) indicate right-sided gene expression. (I) *Nodal* expression in BMP signaling-perturbed embryos (treated from 24 to 48 hpf) at the LG stage. (J) pSmad staining and gene expression patterns in BMP2/4 vMO-treated embryos. The arrow indicates the pSmad-positive HC. (K) The sides of the rudiments (arrows) at the AR stage in BMP2/4 vMO-treated embryos. The numbers in the bottom left-hand corners indicate the ratios of the displayed phenotypes.

Given that pSmad was detected in the HC, we next examined whether BMP signaling is required for HC and rudiment formation. To bypass the early function of BMP signaling in oral-aboral (OA) axial patterning, we used pharmacological treatments to perturb BMP signaling prior to LR axis establishment. A small molecule, dorsomorphin (DM), which selectively inhibits BMP type I receptors and blocks Smad phosphorylation, was previously identified in a screen for compounds that perturb dorsoventral (DV) axis formation in zebrafish [43]. To test whether DM inhibited BMP signaling in the sea urchin, we treated the embryos from fertilization to the mesenchyme stage and performed immunoblot analysis using the pSmad antibody (Figure S3A and Text S1). We observed a dose-dependent reduction of pSmad (Figure S3B). DM also reduced the expression level of *hox7*, a downstream target gene of BMP signaling [33], as well as pSmad staining intensity (Figure S3C). Therefore, DM inhibits BMP signaling in the sea urchin. Conversely, treating embryos with recombinant mouse BMP4 protein (mBMP4) as an exogenous source of BMP ligand expanded *hox7* expression and pSmad signal (Figure S3C). When the embryos were treated with DM or mBMP4 after the OA axis was established (Figure S3H), we observed defects in CP and HC formation. In DM-treated embryos, the levels of *bmp2/4* transcript and pSmad signal were attenuated in the aboral skeletogenic cells at the late gastrula stage (Figure 3A), indicating that *bmp2/4* expression is controlled by its own signaling. The hydropore was not observed in treated pluteus larva, and these embryos also lacked left-sided pSmad staining and ciliated HC (Figure 3B). We observed that the pSmad signal persisted in the aboral skeletogenic and ectodermal cells, indicating that DM did not completely abolish BMP signaling. After the inhibitor was washed out from the culture, 79% of the DM-treated embryos recovered to form rudiments on the left side at the advanced rudiment (AR) stage (Figure 3C). This was significantly lower than that of the controlled embryos (98%). Eighteen percent of the treated embryos formed rudiments on both sides (Figure 3B, bottom panels), and 4% had right-sided rudiments. Ectopic mBMP4 treatment resulted in random positioning of a single undivided CP with *soxE* expression in 89% of the embryos (Figure 3D, open arrowheads). In 11% of mBMP4-treated embryos, two HCs with bilateral *soxE* expression formed (Figure 3D, solid arrowheads, and 3E). These results indicate that BMP signaling is required for proper CP differentiation and left-side HC formation.

We further analyzed LR marker gene expression patterns after BMP signaling was perturbed. The expressions of all examined genes that were normally expressed in the aboral *veg2* descendants, including *soxE*, *pax6*, *six1/2*, *eya*, and *dach*, were diminished in DM-treated embryos but remained in the single CP when BMP signaling was elevated (Figure 3F). Smm genes, such as *vasa, seawi*, *nanos2*, and *foxC*, continued to be expressed at the tip of the archenteron in DM-treated and in the single CP in mBMP4-treated embryos (Figure 3G). These results are in agreement with the lineage analysis demonstrating that BMP signaling acts on aboral *veg2* descendants but not on Smm. Surprisingly, we also discovered that inhibiting and elevating BMP signals both resulted in the loss of right-sided gene expression, including *nodal* and its downstream targets *lefty*, *pitx2*, and *not* (Figure 3H) [6],[39],[44]. The requirement of BMP signals for *nodal* expression is likely indirect because pSmad was not detected in the right lateral ectoderm where *nodal* is expressed (Figure 1C). We further examined *nodal* expression at the late gastrula stage when its right-sided expression began to distinguish whether BMP signals are required for initiation or maintenance of *nodal* expression. The results showed that all embryos lost their right-sided *nodal* expression when BMP signaling was blocked (Figure 3I). The expression of *nodal* either disappeared (84%) or was retained in the oral ectoderm (16%). These results indicate that BMP is required for *nodal* expression initiation, although the mechanism remains unknown.

Although DM has been used as a selective BMP signaling inhibitor [43],[45], it also inhibits a panel of kinases in vitro [46]. Therefore, to specifically inhibit BMP signaling and bypass its early function, we treated the embryos with vivo-morpholinos (vMOs), which are antisense morpholino oligonucleotides linked to eight guanidinium head groups for effective cellular membrane penetration [47]. vMOs have been shown to be effective in a variety of systems, including mice, chick embryo, adult zebrafish, and cultured cells [47]–[49]. We first tested the efficacy and specificity of BMP2/4 vMO in sea urchin embryos. We observed that BMP2/4 vMO effectively blocked green fluorescent protein (GFP) expression in a dose-dependent manner when the embryos were injected with mRNA containing the vMO binding site fused to the GFP sequence (Figure S3D–F and Text S1). The effect was specific because GFP fluorescence was not attenuated by the control or non-specific vMO. When embryos were treated at the 1-cell stage, BMP2/4 vMO also blocked expression of the downstream target *hox7* but had little effect on the non-target *chordin* (Figure S3G, MB). When the vMO was added later at the mesenchyme blastula stage, similar effects were observed (Figure S3G, LG). Therefore, vMOs are effective in the sea urchin embryos and can be used at various developmental stages. When embryos were treated with BMP2/4 vMO from the mesenchyme blastula stage to the late gastrula stage, pSmad staining at the HC disappeared, but *vasa* expression remained in the Smm (Figure 3J). In addition, the expression of *nodal* and the left-sided genes *soxE*, *pax6*, *six1/2*, and *eya* disappeared, which was similar to the effects induced by DM (Figure 3I,J). However, the effects of DM and BMP2/4 vMO on *dach* expression were different (Figure 3F,J). *Dach* expression was absent in DM-treated embryos, but its transcripts remained on the archenteron tip in BMP2/4 vMO-treated embryos. Given that *dach* is expressed in both the oral and aboral archenteron tip at the late gastrula stage (Figure S2B), we suspected that its oral tip expression domain was not affected by BMP2/4 vMO, and the complete disappearance following DM treatment might be due to a non-specific drug effect. We also examined the effects of BMP2/4 vMO on adult rudiment formation. Similar to the effects of DM, most embryos treated with BMP2/4 vMO recovered and formed rudiments on the left side (Figure 3K). A small but significant portion of the treated embryos developed bilateral rudiments, presumably due to the requirement of *nodal* expression by BMP signaling. Taken together, these results supported the hypothesis that BMP signaling is required for proper LR patterning and the expression of the left-sided genes that are normally expressed in aboral *veg2* descendants.

### Nodal Signaling Inhibits BMP Signaling and Its Downstream Target Gene Expression

**Figure 4:**
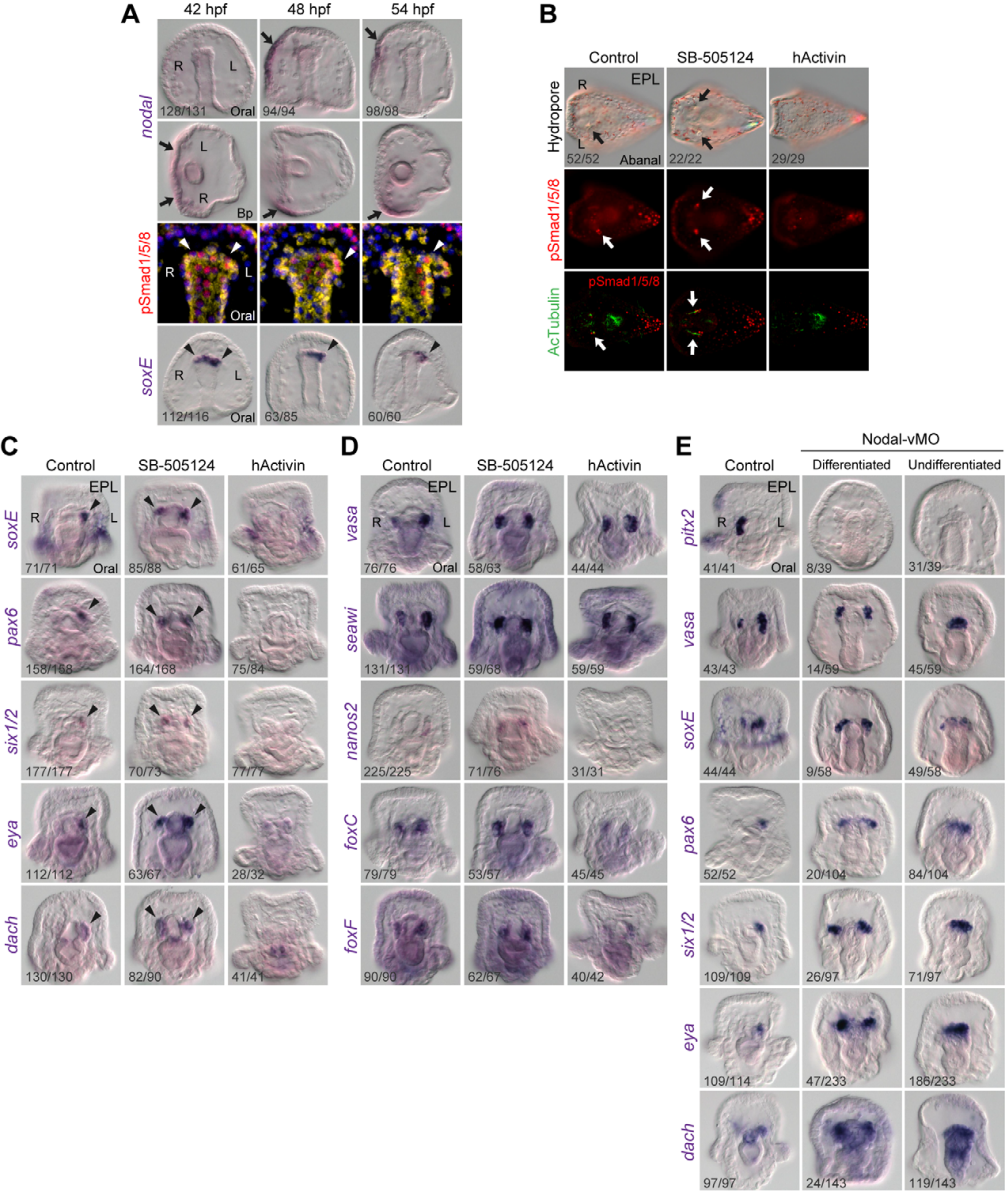
Nodal signaling blocks BMP signaling on the right side. (A) *Nodal* transcripts, pSmad staining, and *soxE* expression in 42, 48, and 54 hpf embryos. The arrows indicate *nodal* expression shifted from oral to the right lateral ectoderm. The arrowheads denote pSmad signal and *soxE* expression that changed from symmetric to the left side at the archenteron tip. (B) pSmad staining in Nodal signaling-perturbed embryos. The arrows indicate the single left HC in control embryos and bilateral HCs in SB-505124-treated embryos. (C and D) LR gene expression patterns after Nodal signals were perturbed. The arrowheads indicate *soxE*, *pax6*, *six1/2*, *eya*, and *dach* expression in the left CP in control and in both CPs in SB-505124-treated embryos. (E) Effects of Nodal vMO on LR marker genes. Note that in 80% of the Nodal vMO-treated embryos, the left and right CPs were not properly divided, and the cells still accumulated on the tip of the archenteron, presumably due to the developmental delay. The numbers in the bottom left-hand corners indicate the ratios of the displayed phenotypes.

Because Nodal is known to function on the right side to prevent the rudiment formation in sea urchins [6], we further examined the relationship between Nodal and BMP signaling during LR axis establishment. At 42 h post-fertilization (hpf), *nodal* expression was in the oral ectoderm and pSmad staining and *soxE* expression were bilateral at the tip of archenteron (Figure 4A). At 48 hpf (late gastrula stage), *nodal* expression shifted to the right lateral ectoderm and pSmad staining and *soxE* transcripts started to restrict on the left side. By 54 hpf, *nodal* expression remained in the right lateral ectoderm, whereas pSmad and *soxE* expression were detected in the differentiating left CP. Therefore, the appearance of *nodal* transcripts on the right side seems to correlate with decreased BMP signaling on the right side. We then examined whether the disappearance of BMP signaling on the right side was regulated by Nodal signals. When Nodal signaling was inhibited with small molecule compounds SB-431542 and SB-505124, pSmad staining became bilateral, and two ciliated HCs also formed, which resulted in two hydropores on the aboral ectoderm (Figures 4B and S4A, arrows). In addition, elevating Nodal signaling with recombinant human Activin (hActivin) prevented HC formation. These results suggest that Nodal signals are upstream of BMP signaling and normally block BMP action on the right side.

We further analyzed the effects of Nodal signaling on LR-related genes. When Nodal signaling was blocked, *nodal*, *lefty*, *pitx2*, and *not* were downregulated, confirming that Nodal signaling is upstream of the right-sided genes (Figure S4B). However, enhancing Nodal signaling with hActivin did not lead to bilateral expression of right-sided genes. This result is different from the previous study in *P. lividus* sea urchin [6], suggesting that the transcriptional regulation of the *nodal* gene might differ in the two species. Consistent with the idea that Nodal signals block BMP signaling, we observed that all left-sided BMP signaling downstream target genes were expressed bilaterally in both CPs when Nodal signaling was blocked, whereas hActivin inhibited their expression (Figures 4C and S4C, arrowheads). Interestingly, Nodal signaling had different effects on genes that are expressed in the Smm. The expression of *nanos2* became bilateral when Nodal signaling was blocked. In the hActivin-treated embryos, *nanos2* expression was diminished, and *foxC* and *foxF* transcripts were reduced. The LR asymmetric *vasa* and *seawi* signals became symmetric when Nodal signaling was blocked (Figures 4D and S4D). Similar effects were also observed when the embryos were treated with Nodal vMO (Figures 4E and S4E). These results suggest that right-sided Nodal signaling represses *nanos2* expression and controls the asymmetric expression of *vasa* and *seawi*.

### Nodal Signaling Regulates Asymmetrical Separation and Induces Small Micromere Apoptosis

**Figure 5:**
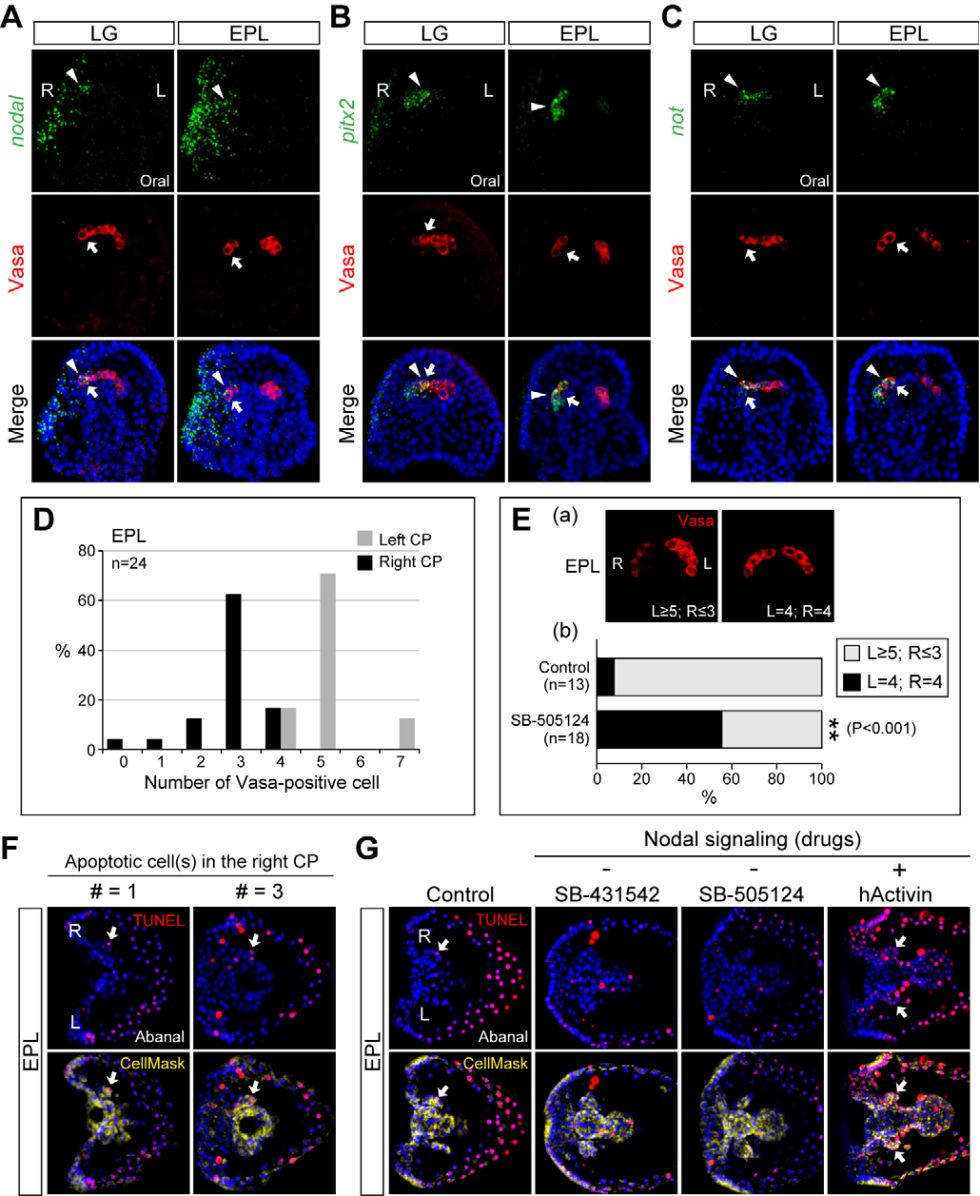
Nodal controls the asymmetric separation and apoptosis of small micromeres. ISH of *nodal* (A), *pitx2* (B), and *not* (C) demonstrate that they are expressed in Smm on the right side (arrowheads) that are marked by Vasa antibody (arrows). (D) Percentages of Vasa-positive Smm in the left and right CPs. (E) The asymmetric and symmetric separation of Vasa-positive Smm (a) and the percentages (χ*^2^* test) in control and SB-505124-treated embryos (b). (F) TUNEL staining revealed one (# = 1) to three (# = 3) apoptotic cells in the right CP. (G) TUNEL staining after perturbing Nodal signaling. The arrows in (F) and (G) indicate apoptotic cells in the CPs.

Although we showed that Nodal signaling prevented left-sided development by inhibiting BMP signaling in *veg2* descendants, the effects of Nodal signaling on the Smm are unclear. A detailed analysis of *nodal* and *pitx2* transcripts revealed that in addition to the expression in the right lateral ectoderm, both genes were expressed in the Vasa-positive Smm in the right CP at the late gastrula and the pluteus stages (Figure 5A,B). Another Nodal downstream gene, *not*, exhibited a similar expression pattern, except that it was not detected in the ectoderm (Figure 5C). These data suggest that Nodal signaling is received by the Smm on the right side. At the early pluteus stage, we frequently observed that BrdU- or Vasa-positive Smm were asymmetrically partitioned into the left and right CPs and eventually disappeared from the right side. Of the eight Smm at this stage, in most cases, five and three cells were partitioned into the left and right CP, respectively (Figure 5D). This 5∶3 ratio was also observed when Smm were labeled with histone H1cs antiserum [50]. Because the right-sided Smm receive Nodal signals, we next determined whether Nodal signaling controls their asymmetrical separation. We classified the separation as asymmetrical (L≥5; R≤3) or symmetrical (L = 4; R = 4) (Figure 5Ea). Inhibiting Nodal signaling significantly increased the rate of symmetrical separation from 8% to 56% (Figure 5Eb), indicating that Nodal signaling regulates asymmetrical Smm separation. These results are consistent with the observation that the asymmetric expression patterns of Smm-expressed genes, such as *vasa* and *seawi*, became symmetric after Nodal signaling was blocked (Figure 4D,E).

When labeling Smm with the Vasa antibody, we observed that the number of the Vasa-positive Smm on the right side decreased to two or even one in some of the pluteus larva (Figure 5D). This observation suggests that the disappearance of the Smm on the right side is a gradual process. It was proposed that *nanos*-depleted Smm undergo apoptosis [51],[52]. Because *nanos2* expression was blocked by Nodal signaling in the right-sided Smm (Figure 4D), we wondered whether Nodal signaling induces Smm to undergo apoptosis. When the larvae were stained with TUNEL, we observed that one to three cells in the right CP were apoptotic, whereas no apoptotic cells were detected in the left CP (Figure 5F). Furthermore, inhibition of Nodal signaling prevented apoptosis in the CPs, and hActivin treatment resulted in apoptotic cells in both CPs (Figure 5G, arrows). These effects correlate well with *nanos2* expression following Nodal signaling perturbation. These data suggest that Nodal signaling induces apoptosis in the right-sided Smm, possibly by controlling *nanos2* expression. In addition to apoptotic cells in the right CP, we also observed TUNEL-positive cells in the aboral ectoderm of pluteus larva (Figure 5G). These signals were attenuated and increased when Nodal signaling was blocked and elevated, respectively, suggesting that Nodal signaling is also involved in aboral ectodermal cell apoptosis.

### The Molecular Pathways in LR Patterning

**Figure 6:**
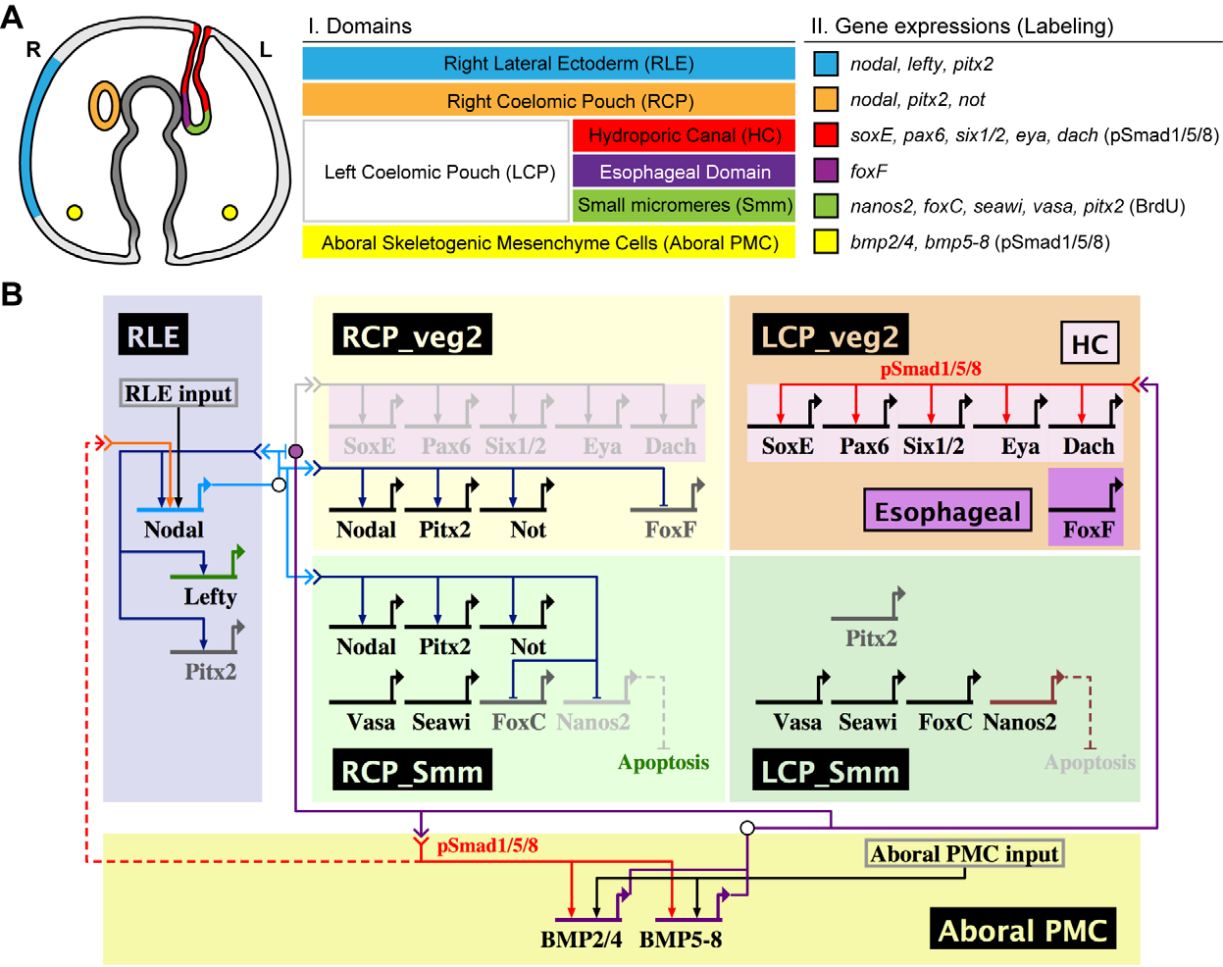
Summary of gene expression domains and molecular pathways involved in LR patterning. (A) Illustration of gene expression domains in the early pluteus larva (oral view). Different colors represent different domains (I) that express a different set of genes (II), as indicated. (B) Schematic representation of the molecular pathways in LR patterning, created using the BioTapestry program [69]. BMP signal from the aboral skeletogenic mesenchyme cells (aboral PMC) is required for *nodal* expression in the right lateral ectoderm (RLE) through an unknown mechanism (red dashed line). Nodal signal at the RLE activates its downstream genes in the RLE and right CP (RCP). Nodal signal also induces apoptosis in the right-sided Smm and presumably does so by repressing *nanos2* expression. Right-sided BMP signaling is blocked by Nodal and only functions in aboral *veg2* descendants in the left CP (LCP) to activate its downstream genes. The genes shown in black, light gray, or dark gray are expressed, not expressed, or expressed weakly, respectively, in their specific domains at the early pluteus stage. The arrows do not necessarily indicate direct interactions.

Based on the lineage and perturbation analyses, we provided a schematic representation of the molecular pathways in LR patterning. Figure 6 represents the relationships between Nodal and BMP signals in controlling genes expressed in the right or left CP from two lineages, Smm and *veg2* descendants, at the early pluteus stage. We showed that although *bmp* genes are expressed in aboral skeletogenic cells, pSmad staining was detected in the left-sided HC at the pluteus stage. These cells express *soxE*, *pax6*, *six1/2*, *eya*, and *dach*. The esophageal domain of the left CP expresses *foxF*. The initial bilateral pSmad signal at the tip of the archenteron in the late gastrula stage becomes restricted to the left side due to the inhibition by right-sided Nodal signaling, which also regulates its downstream genes in the right CP. Moreover, the initiation of *nodal* expression on the right side indirectly depends on BMP signaling, and a right lateral ectoderm (RLE) input may also be involved in the spatial regulation of *nodal* expression. Taken together, these data suggest that BMP signaling is both upstream and downstream of Nodal signaling.

## Discussion

Most sea urchin adult tissues derive from the rudiment developed from the left CP. Although it is known that both Smm and the *veg2* mesoderm contribute to the CPs, past studies were unable to clearly identify genes that are specifically expressed in either lineage. It was also unknown which of the two lineages contributed to the left CP-derived HC. In addition to identifying several lineage-specific genes in the CP and the HC, we also provided evidence to demonstrate that BMP signals act in the left CP together with Nodal signaling to regulate LR patterning. Given that left-sided *nodal* expression is a conserved feature in chordates [53],[54] and right-sided BMP signaling is observed in several vertebrate species, the opposing Nodal and BMP signals regulating LR asymmetry is likely a conserved mechanism in deuterostomes. However, the mechanisms controlling LR asymmetry in the sea urchin are reversed compared to chordates, using the convention that the mouth is located on ventral sides of embryos. Thus, our study reinforces the possibility that DV inversion occurred in the chordate lineage. Below, we discuss other important findings from this study.

### Opposing Nodal and BMP Signals Control LR Axis Patterning

We demonstrated that elevating either Nodal or BMP signaling resulted in the loss of the other signal. This mutual antagonism between Nodal and BMP signaling has been observed during LR patterning in vertebrates. Nodal signaling inhibits BMP signals in the left LPM of mouse embryos by activating the expression of *chordin* and *noggin* genes, which encode BMP antagonists [10]. BMP signaling also has been shown to block Nodal signals in the right LPM of mouse, chick, and zebrafish embryos by activating the expression of *lefty* genes that encode Nodal antagonists [8],[55]. The inhibition of BMP signals by Nodal signaling has also been observed in sea urchin embryos during DV (aboral-oral) axis establishment. Nodal signaling in the oral ectoderm is required for the expression of *chordin*, which restricts BMP signals in the aboral ectoderm [31]. However, we could not detect any asymmetrical LR expression of genes encoding BMP antagonists, such as *chordin*, *noggin*, *follistatin*, *dan*, or *gremlin* in the sea urchin embryo (unpublished data). The second molecular mechanism to explain the mutual antagonism between Nodal and BMP signaling is the direct competition between the two signals for the limited amount of the common effector Smad4. In the mouse embryo, BMP signaling has been shown to set a repressive threshold for Nodal signaling in the LPM by limiting Smad4 availability [56].

### Micromere-Derived Signals Control LR Asymmetry in Sea Urchin Embryos

The repressive role of Nodal signaling on BMP in the sea urchin embryo is evident given that increasing or blocking Nodal signaling results in the loss of or bilateral pSmad staining in CPs, respectively. However, the effects of BMP signaling on Nodal are complicated because increasing and blocking BMP signaling both result in the loss of *nodal* expression. These results suggest that BMP signaling is required for right-sided *nodal* expression in the sea urchin embryo. This positive role of BMP signaling on *nodal* gene expression has also been observed in vertebrates. In the absence of mouse embryonic BMP4, *nodal* expression is lost in the left LPM [57]. In chick embryos, implanting either *bmp2*-expressing cells [58] or BMP-soaked beads [59] in the LPM increases *nodal* expression. During the late segmentation stages of zebrafish embryos, BMP4 signaling is required to activate the expression of the *nodal*-related gene *cyclops* in the left LPM [60]. Therefore, BMP signaling can be a positive or negative regulator of Nodal signaling depending on the developmental stages and tissue layers during LR patterning in vertebrates.

Although we observed LR asymmetrical BMP signaling with pSmad staining in the CPs in the sea urchin, *bmp* genes are transcribed in the skeletogenic mesenchyme cells near the aboral apex of the larva. These observations suggest that BMP ligands are secreted from these micromere-derived skeletogenic cells to control LR asymmetry. It was previously shown that when the micromere lineage was removed from embryos of sea urchin *Hemicentrotus pulcherrimus*, the LR placement of the rudiment was randomized [26]. BMP could be the micromere-derived signal that regulates larval LR polarity, although other signaling molecules may also be involved in this process.

### Nodal, Nanos, and Apoptosis in Small Micromeres

In sea urchin embryos, Smm form during the fifth cleavage and are regulated by a set of conserved germline lineage genes, including *vasa*, *nanos2*, and *seawi* [37]. Adults from Smm-deleted embryos formed small gonads without gametes [61]. Therefore, Smm are required for germline specification in sea urchins. Smm lineage fate is maintained by Nanos [52], which is also required for Smm descendant survival [51]. Studies in fly and vertebrates have also shown that Nanos has a conserved role in maintaining germline identity by preventing apoptosis [62]–[64]. In this study, we showed that Nodal signaling acts on the Smm partitioned in the right CP. Nodal signaling perturbation affected both *nanos2* expression and cell death. These results suggest that Nodal signaling in the right CP represses *nanos2* expression in the Smm, which leads to cell death. To our knowledge, the role of Nodal signaling in repressing the expression of germline lineage genes such as *nanos* has not been reported in other systems. Intriguingly, Nodal is capable of inducing apoptosis in adult ovary during follicular degeneration and human trophoblast cells during normal placentation [65],[66]. Therefore, there may be a conserved role for Nodal to induce apoptosis in extraembryonic tissues. In the case of sea urchins, Nodal signaling-induced apoptosis in the right Smm is essential for normal development, and a lack of Nodal results in bilateral rudiments that give rise to a juvenile composed of two conjoined urchins [6].

## Materials and Methods

### Animals, Embryos, and Treatments

Adult sea urchins (*Strongylocentrotus purpuratus*) and their gametes were obtained as previously described [29]. *Rhodomonas lens* provided by Pat Leahy was used to feed the larva (see Text S1). BMP or Nodal signaling perturbation was performed by treating embryos with inhibitors, recombinant proteins, or vivo-morpholinos (vMOs) and culturing them in the dark. Unless otherwise indicated, the concentrations of the reagents used in this study were as follows: dorsomorphin (16 µM; Sigma), SB-431542 (5 µM; Tocris Cookson), SB-505124 (2 µM; Sigma), mouse BMP4 (250 ng/ml; R&D Systems), and human Activin AB (120 ng/ml; R&D systems). Solvents (DMSO or 0.1% BSA) were added as controls. The sequences of the BMP and Nodal vMOs (Gene Tools) are the same as the previously published regular MOs [33]. vMOs were diluted at 1∶100 to 5 µM from stock solution in phosphate-buffered saline into 500 µl of culture. The treatment times are summarized in Figures S3H and S4E. To keep the larva feeding normally and viable, the treatments were washed out no later than EPL stage (72 hpf). Note that at higher concentrations (over 20 µM), the vMOs precipitate in seawater and are toxic to the embryos.

### In Situ Hybridization and Immunostaining

The primers used to construct the clones for probe synthesis were designed based on gene models [67] and are listed in Table S1. In situ hybridization (ISH) and immunostaining were performed as previously described [29]. The primary antibodies used in this study were rabbit anti-pSmad1/5/8 (1∶200; Cell Signaling), mouse anti-acetylated α-tubulin (1∶1,000; Sigma), and rabbit anti-DmVasa (1∶300) [68]. The nuclei were counterstained with Hoechst 33342 (1∶1,000; Invitrogen), and the cytoplasmic membrane was visualized with CellMask Deep Red (1∶2,000; Invitrogen). The embryos were imaged using a Leica TCS-SP5 AOBS inverted confocal system.

### BrdU Labeling and TUNEL Assay

After removing the fertilization envelope, 1-cell-staged embryos were incubated with 50 µM 5-bromo-2-deoxyuridine (BrdU; Sigma) for 1 h and then washed twice with 500 µM thymidine. For double labeling, the biotin-avidin system was used to detect the BrdU signal. Antigen retrieval of the incorporated BrdU was conducted by DNA denaturation using 1 N HCl in PBST for 30 min. To block endogenous biotin, the embryos were incubated with 0.01% avidin and 0.001% biotin sequentially. The embryos were then immunolocalized with biotinylated anti-BrdU antibody (1∶1,000; Abcam) and detected with HiLyte Fluor Streptavidin (1∶400; AnaSpec). Terminal deoxynucleotidyl transferase dUTP nick end labeling (TUNEL) was performed by using the In Situ Cell Death Detection Kit (TMR red; Roche) for 40 min at 37°C.

## Supporting Information

Figure S1 Developmental processes and LR asymmetry in the sea urchin. (A) Schematic illustrations of developmental processes from radial symmetric blastula, bilateral symmetric gastrula, left-right asymmetric larva, to pentasymmetric body plan. At the end of gastrulation, two coelomic pouches (CPs) form on each side of the archenteron tip. A distinct LR asymmetry occurs when the hydroporic canal (HC) evaginates from the left CP. The CPs then divide into the axocoel (ax), hydrocoel (hy), and somatocoel (so). The invaginated left oral ectoderm forms the primary vestibule (pv), which makes contact with the enlarged left hydrocoel to form an adult rudiment (ru) on the left side of the larva. A secondary vestibule (sv) also invaginates from the right oral ectoderm. (B–M) LR asymmetry in the sea urchin. At the early pluteus stage (EPL, 72 hpf), the HC is formed on the left side (B) with a hydropore (hp) opening on the left aboral ectoderm (C). (D) Higher magnification of the HC in the four-arm larva (E). (F) The HC is the first morphological sign of LR asymmetry. (G) Vestibular invagination stage larva. (H) The vestibule and the left hydrocoel become apposed (arrow) at the rudiment initiation stage. (I–J) At the pentagonal disc stage, the five tube-foot primordia (tfp) project against the vestibular floor (arrow). (K) At the advanced rudiment stage, the rudiment with adult plates forms on the left side of the larva. (L) Higher magnification of the juvenile spines (js) that develop at the posterior apex and on the right side of the larva. (M) A juvenile sea urchin with juvenile spines and adult spines (as) after metamorphosis. The observed view is indicated in the bottom right-hand corner of each panel, and the axes are labeled as O, oral; A, aboral; L, left; and R, right.
(TIF)

Figure S2 Cell lineage analysis and gene expression patterns during CP formation. (A) Smm were labeled with BrdU and Vasa antibody at the LG and EPL stages. (B) Whole mount ISH of *soxE*, *pax6*, *six1/2*, *eya*, and *dach* at the LG and EPL stages. (C) Double fluorescence ISH analyses of *soxE* with *pax6*, *six1/2*, *eya*, or *dach* at the EPL stage. (D) Expression patterns of the germline marker genes at the LG and EPL stages. (E) Expression patterns of *foxC* and *foxF* at the LG and EPL stages. (F) Double fluorescence ISH analyses of *foxC* and *nanos2* or *foxF*. (G) Expression of *foxY* at different developmental stages. The solid arrowheads indicate Smm, the arrows indicate signals in the aboral tip and HC, and the open arrowheads denote expression in the oral tip of the archenteron and the esophageal domain.
(TIF)

Figure S3 Efficacy of dorsomorphin and vMOs. (A) Western blot analysis using anti-pSmad antibody on mesenchyme blastula (MB) extracts revealed a major band around 60 kDa as predicted (arrow). (B) The pSmad levels decreased upon dorsomorphin treatment. β-tubulin was used as a loading control to quantify the pSmad1/5/8 protein levels. (C) Expression of *hox7* and pSmad signals in the BMP signaling-perturbed embryos. (D) The embryos were incubated with the vMOs after injecting with the mRNA containing vMO-binding sites upstream of the GFP coding sequence (constructs shown on the right). The effects of the vMOs on the fluorescence intensities of GFP were quantified in embryos treated with different concentrations of the vMOs after injecting GFP mRNA containing the BMP2/4 (E) or Nodal (F) vMO-binding site. Error bars are standard errors of the mean. (G) ISH of *chordin* and *hox7* in 5 µM BMP2/4 vMO- or Nodal vMO-treated embryos. The upper panels (MB) were treated from one-cell to MB stage. The bottom panels (LG) were treated from MB to LG stage. The numbers in the bottom left-hand corners of the photos indicate the phenotype ratios. (H) The embryos were incubated for the indicated time and concentration, and the effects on the HC or CP were assessed. The listed effect represents the phenotype observed in over 90% of embryos. The black bar shows the treatment time used in most experiments. Note that the effective timing for DM and vMO treatment was different (H). DM treatment from 42 to 48 hpf was sufficient to block HC formation, whereas the vMO had no effect when applied in the same period. The different effects might be due to the natures of the two blocking mechanisms. DM inhibits BMP receptor kinase activity and blocks BMP signaling immediately after it penetrates cells. On the other hand, the vMO blocks translation of *bmp2/4*, and BMP signaling could still be active until the remaining BMP2/4 is degraded.
(TIF)

Figure S4 Effects of Nodal signaling on LR asymmetry. (A) pSmad and acetylated α-tubulin staining in SB-431542-treated embryos revealed bilateral HC (arrows) in EPL. (B) Expression of right-sided genes (indicated by arrows) following Nodal signaling perturbation. (C and D) Expression of LR marker genes after SB-431542 treatments. The numbers in the bottom left-hand corners of the photos indicate the phenotype ratios. (E) The embryos were incubated for the indicated time and concentration, and the effects on the oral-aboral (OA) axis and HC formation in over 90% of the embryos are listed. The black bar shows the treatment time used in most experiments. The effect of SB inhibitors and Nodal vMO was different in that Nodal vMO did not cause OA defects when treated during MB. The difference might also be due to the differential inhibitory mechanisms: SB inhibitors directly block signaling, whereas vMO blocks translation of the ligand.
(TIF)

Table S1 Gene IDs and primers used to construct clones for probe synthesis in this study.
(XLS)

Text S1 Supplementary methods.
(DOC)

## Abbreviations

BMP: bone morphogenetic protein
CP: coelomic pouch
DM: dorsomorphin
HC: hydroporic canal
LR: left-right
RLE: right lateral ectoderm
Smm: small micromeres
vMOs: vivo-morpholinos
