# Supplementary figures and images for: Opposing Nodal and BMP Signals Regulate Left–Right Asymmetry in the Sea Urchin Larva
Source: PLoS Biol. 2012 Oct 9;10(10):e1001402. doi: 10.1371/journal.pbio.1001402 (PMC3467216; doi:10.1371/journal.pbio.1001402)

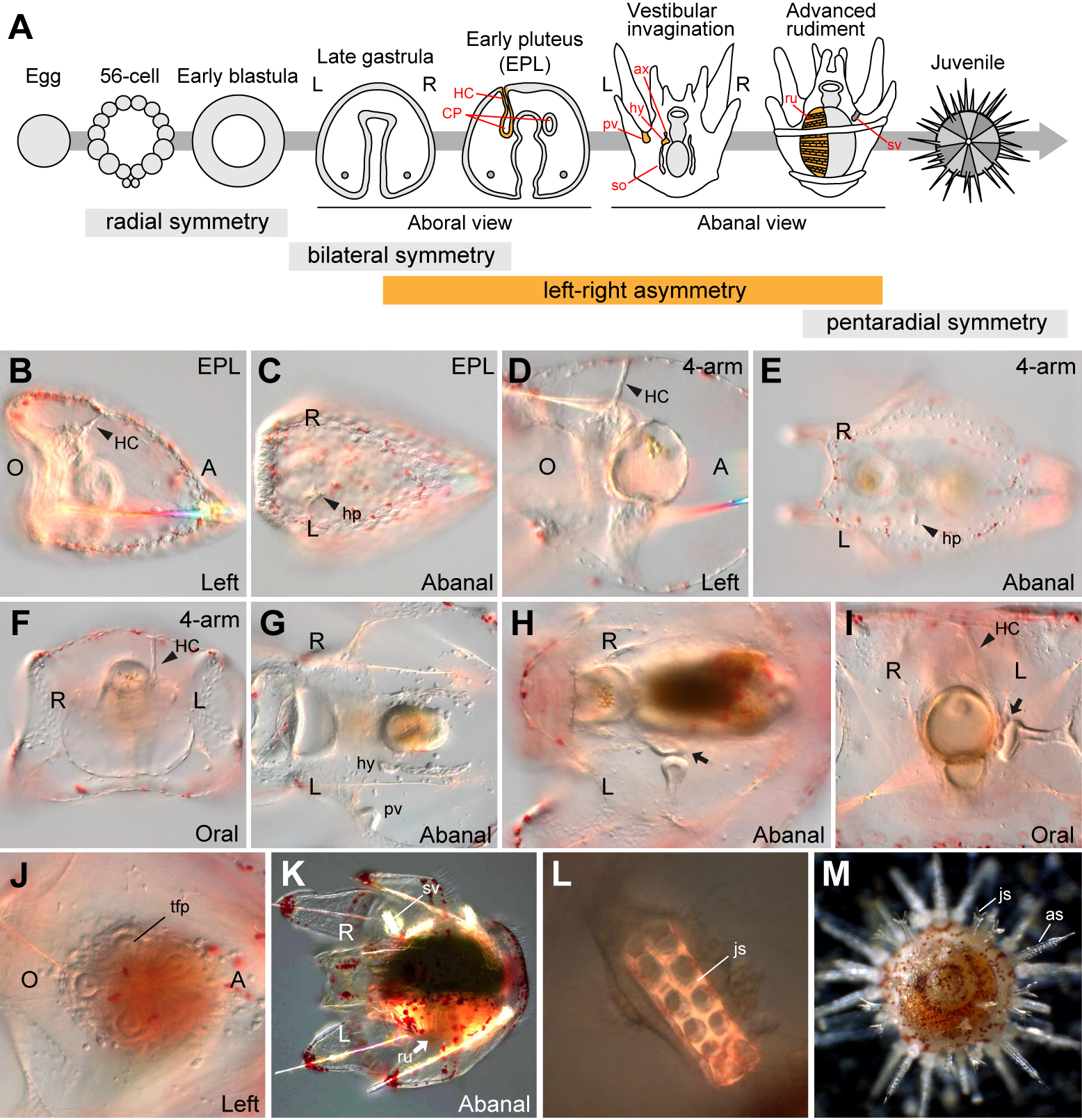

Supplement: Figure S1 — Developmental processes and LR asymmetry in the sea urchin. (A) Schematic illustrations of developmental processes from radial symmetric blastula, bilateral symmetric gastrula, left-right asymmetric larva, to pentasymmetric body plan. At the end of gastrulation, two coelomic pouches (CPs) form on each side of the archenteron tip. A distinct LR asymmetry occurs when the hydroporic canal (HC) evaginates from the left CP. The CPs then divide into the axocoel (ax), hydrocoel (hy), and somatocoel (so). The invaginated left oral ectoderm forms the primary vestibule (pv), which makes contact with the enlarged left hydrocoel to form an adult rudiment (ru) on the left side of the larva. A secondary vestibule (sv) also invaginates from the right oral ectoderm. (B–M) LR asymmetry in the sea urchin. At the early pluteus stage (EPL, 72 hpf), the HC is formed on the left side (B) with a hydropore (hp) opening on the left aboral ectoderm (C). (D) Higher magnification of the HC in the four-arm larva (E). (F) The HC is the first morphological sign of LR asymmetry. (G) Vestibular invagination stage larva. (H) The vestibule and the left hydrocoel become apposed (arrow) at the rudiment initiation stage. (I–J) At the pentagonal disc stage, the five tube-foot primordia (tfp) project against the vestibular floor (arrow). (K) At the advanced rudiment stage, the rudiment with adult plates forms on the left side of the larva. (L) Higher magnification of the juvenile spines (js) that develop at the posterior apex and on the right side of the larva. (M) A juvenile sea urchin with juvenile spines and adult spines (as) after metamorphosis. The observed view is indicated in the bottom right-hand corner of each panel, and the axes are labeled as O, oral; A, aboral; L, left; and R, right. (TIF) [file pbio.1001402.s001.tif]

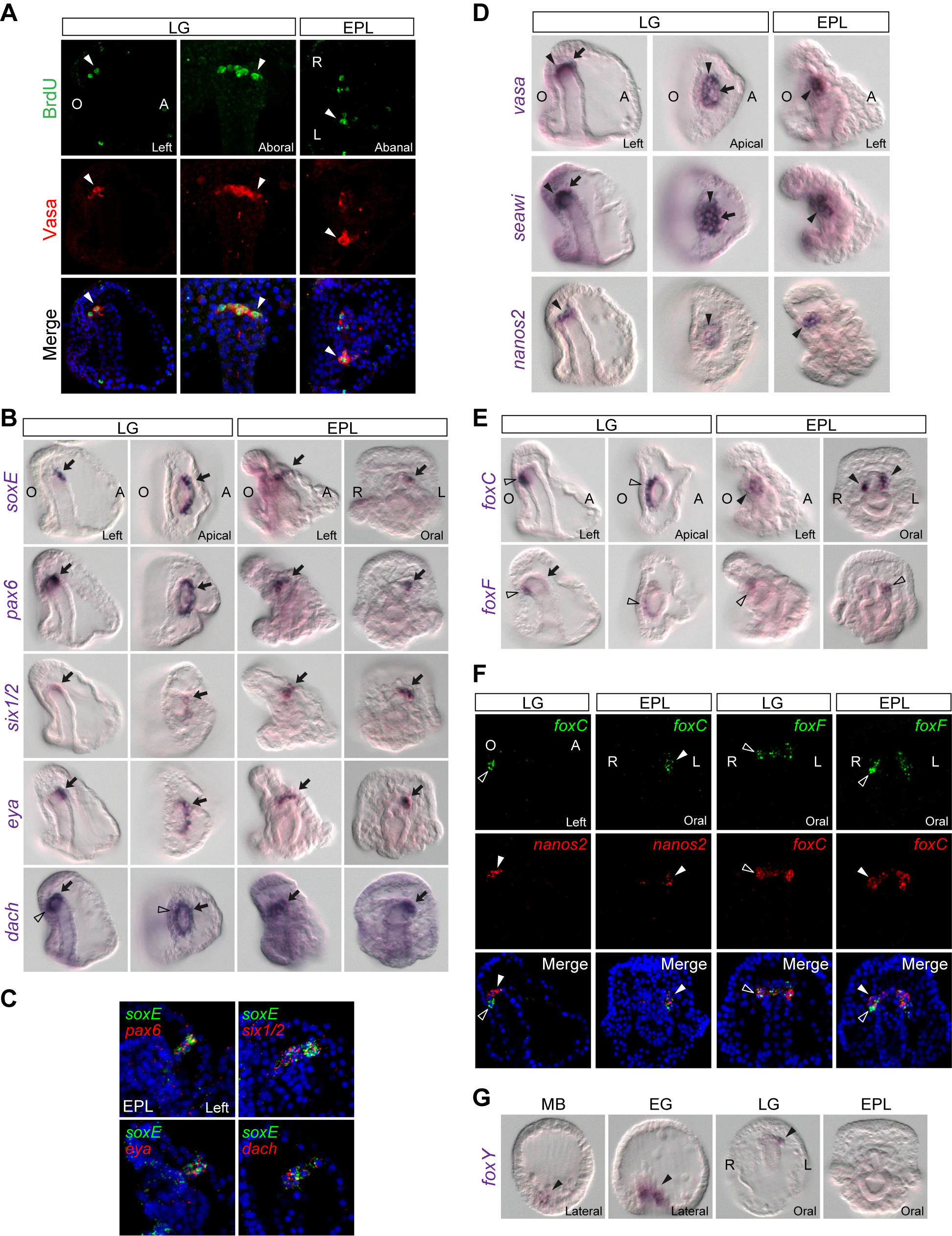

Supplement: Figure S2 — Cell lineage analysis and gene expression patterns during CP formation. (A) Smm were labeled with BrdU and Vasa antibody at the LG and EPL stages. (B) Whole mount ISH of soxE, pax6, six1/2, eya, and dach at the LG and EPL stages. (C) Double fluorescence ISH analyses of soxE with pax6, six1/2, eya, or dach at the EPL stage. (D) Expression patterns of the germline marker genes at the LG and EPL stages. (E) Expression patterns of foxC and foxF at the LG and EPL stages. (F) Double fluorescence ISH analyses of foxC and nanos2 or foxF. (G) Expression of foxY at different developmental stages. The solid arrowheads indicate Smm, the arrows indicate signals in the aboral tip and HC, and the open arrowheads denote expression in the oral tip of the archenteron and the esophageal domain. (TIF) [file pbio.1001402.s002.tif]

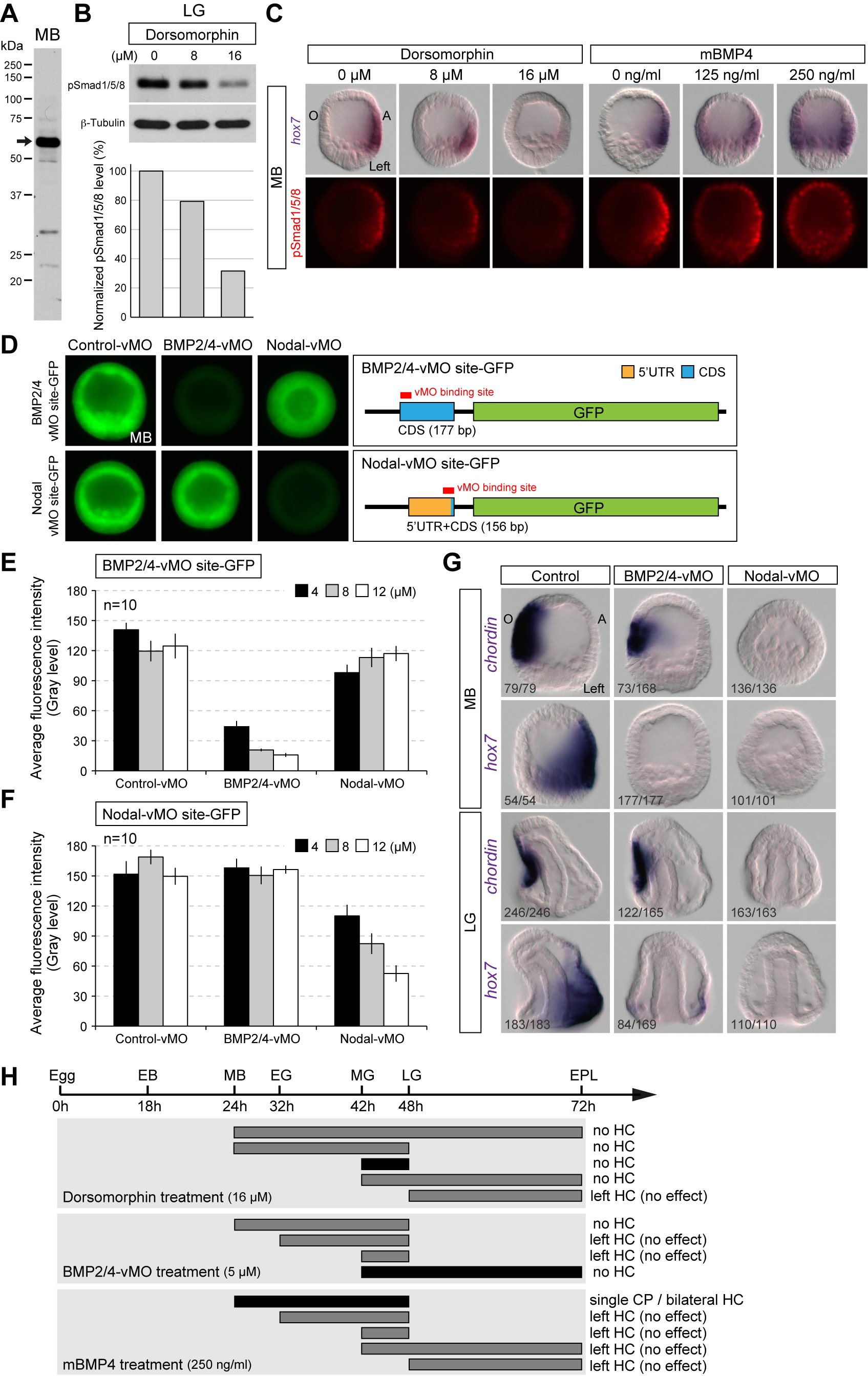

Supplement: Figure S3 — Efficacy of dorsomorphin and vMOs. (A) Western blot analysis using anti-pSmad antibody on mesenchyme blastula (MB) extracts revealed a major band around 60 kDa as predicted (arrow). (B) The pSmad levels decreased upon dorsomorphin treatment. β-tubulin was used as a loading control to quantify the pSmad1/5/8 protein levels. (C) Expression of hox7 and pSmad signals in the BMP signaling-perturbed embryos. (D) The embryos were incubated with the vMOs after injecting with the mRNA containing vMO-binding sites upstream of the GFP coding sequence (constructs shown on the right). The effects of the vMOs on the fluorescence intensities of GFP were quantified in embryos treated with different concentrations of the vMOs after injecting GFP mRNA containing the BMP2/4 (E) or Nodal (F) vMO-binding site. Error bars are standard errors of the mean. (G) ISH of chordin and hox7 in 5 µM BMP2/4 vMO- or Nodal vMO-treated embryos. The upper panels (MB) were treated from one-cell to MB stage. The bottom panels (LG) were treated from MB to LG stage. The numbers in the bottom left-hand corners of the photos indicate the phenotype ratios. (H) The embryos were incubated for the indicated time and concentration, and the effects on the HC or CP were assessed. The listed effect represents the phenotype observed in over 90% of embryos. The black bar shows the treatment time used in most experiments. Note that the effective timing for DM and vMO treatment was different (H). DM treatment from 42 to 48 hpf was sufficient to block HC formation, whereas the vMO had no effect when applied in the same period. The different effects might be due to the natures of the two blocking mechanisms. DM inhibits BMP receptor kinase activity and blocks BMP signaling immediately after it penetrates cells. On the other hand, the vMO blocks translation of bmp2/4, and BMP signaling could still be active until the remaining BMP2/4 is degraded. (TIF) [file pbio.1001402.s003.tif]

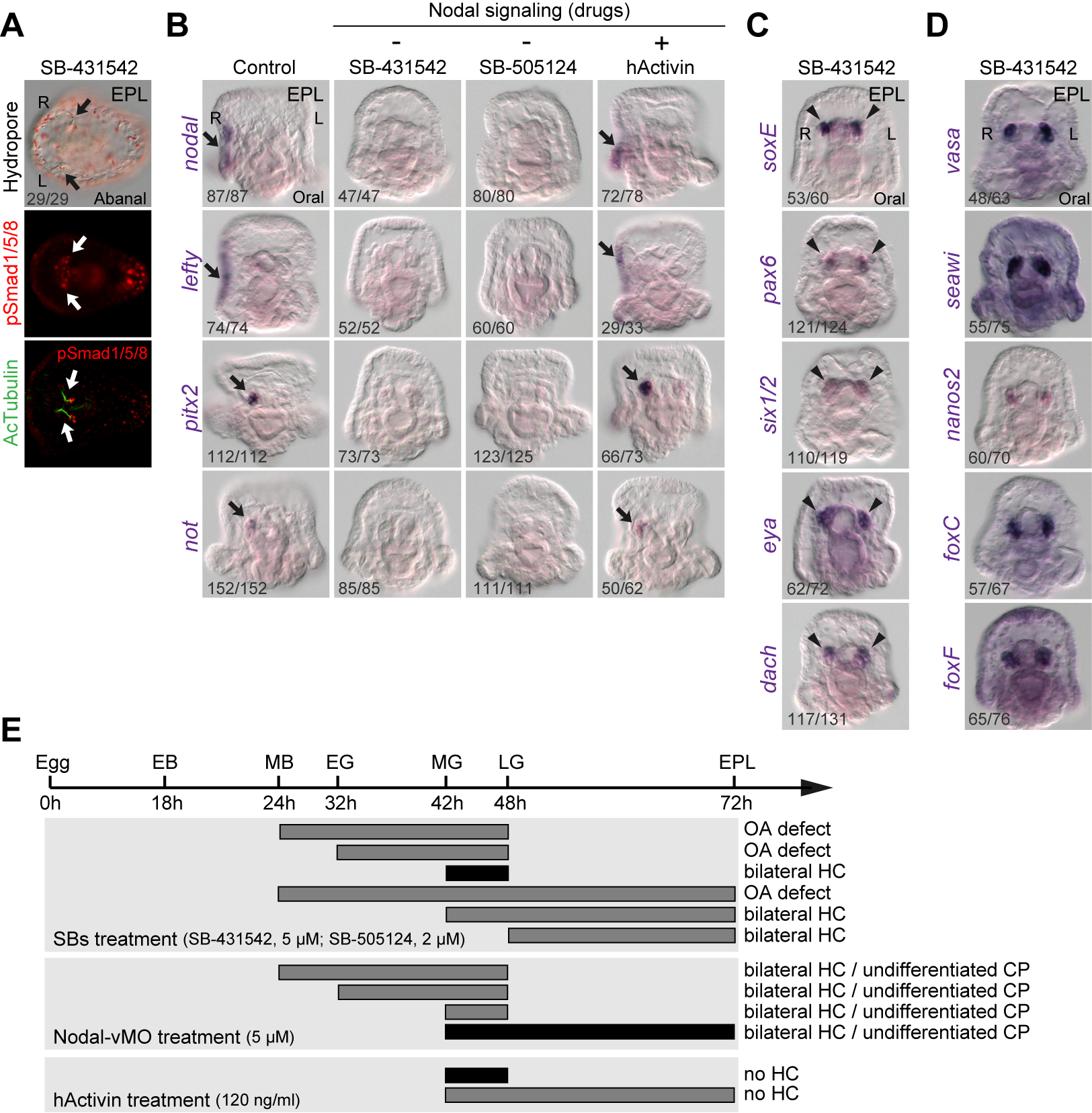

Supplement: Figure S4 — Effects of Nodal signaling on LR asymmetry. (A) pSmad and acetylated α-tubulin staining in SB-431542-treated embryos revealed bilateral HC (arrows) in EPL. (B) Expression of right-sided genes (indicated by arrows) following Nodal signaling perturbation. (C and D) Expression of LR marker genes after SB-431542 treatments. The numbers in the bottom left-hand corners of the photos indicate the phenotype ratios. (E) The embryos were incubated for the indicated time and concentration, and the effects on the oral-aboral (OA) axis and HC formation in over 90% of the embryos are listed. The black bar shows the treatment time used in most experiments. The effect of SB inhibitors and Nodal vMO was different in that Nodal vMO did not cause OA defects when treated during MB. The difference might also be due to the differential inhibitory mechanisms: SB inhibitors directly block signaling, whereas vMO blocks translation of the ligand. (TIF) [file pbio.1001402.s004.tif]
