# Supplementary material for: Opposing Nodal and BMP Signals Regulate Left–Right Asymmetry in the Sea Urchin Larva
Source: PLoS Biol. 2012 Oct 9;10(10):e1001402. doi: 10.1371/journal.pbio.1001402 (PMC3467216; doi:10.1371/journal.pbio.1001402)
Supplement: Text S1 — Supplementary methods. (DOC) [file pbio.1001402.s006.doc]

**Text S1 Supplementary Methods**

**Culture and Observation of Larvae**

The larvae were reared in 250 ml beakers with filtered seawater (FSW) in a photoperiod 14/10 (light/dark) and fed with *Rhodomonas lens*. Agitation was applied at 50 rpm using a handmade rotator. The FSW was changed once a week, and the larvae were fed twice a week. For observing live larvae at the late pluteus stage, the larvae were anesthetized with 10% ethanol in FSW for 10 minutes.

**Western Blot Analysis**

Embryos were lysed in lysis buffer (20 mM Tris-HCl, 150 mM NaCl, 1% Triton X-100, pH 8) with a protease inhibitor cocktail (Sigma) and phosphatase inhibitors (50 mM NaF, 1 mM Na_3_VO_4_, 1 mM Na_4_P_2_O_7_, 1 mM β-Glycerophosphate) for 30 minutes at 4°C. The lysates were obtained after centrifugation at 10,000x*g* for 20 minutes. The concentrations of proteins were determined by BCA assay (Thermo). The protein extracts were resolved using 10% SDS-PAGE and then transferred to PVDF membranes for Western blot analysis. Anti-pSmad1/5/8 antibody was used in a 1:2000 dilution followed by anti-rabbit HRP secondary antibody diluted to 1:50000 (Jackson ImmunoResearch). The signal was detected using SuperSignal West Dura Extended Duration Substrate (Thermo). For the loading control, the membrane was stripped and incubated with anti-β-tubulin antibody (1:5000; Sigma).

**Microinjection of mRNA and vMO Incubation**

The effectiveness and specificity of the vivo-morpholinos (vMOs; Gene Tools) were assessed by injecting the mRNA (0.1 mg/ml) containing the vMO target sequence fused in-frame to GFP. The injected embryos were then incubated with vMOs. Sequences of vMOs were: BMP2/4 vMO, 5’-ACCCCAATGTGAGGTGGTAACCAT-3’; Nodal vMO, 5’-TGCATGGTTAAAAGTCCTTAAAAAT-3’. The standard control vMO was also from Gene Tools. For early treatments, the fertilization envelope was removed, and the fertilized eggs were directly treated with vMO in FSW.
